# Supplementary figures and images for: Modulation of the Translation Efficiency of Heterologous mRNA and Target Protein Stability in a Plant System: The Case Study of Interferon-αA
Source: Plants (Basel). 2022 Sep 20;11(19):2450. doi: 10.3390/plants11192450 (PMC9573741; doi:10.3390/plants11192450)

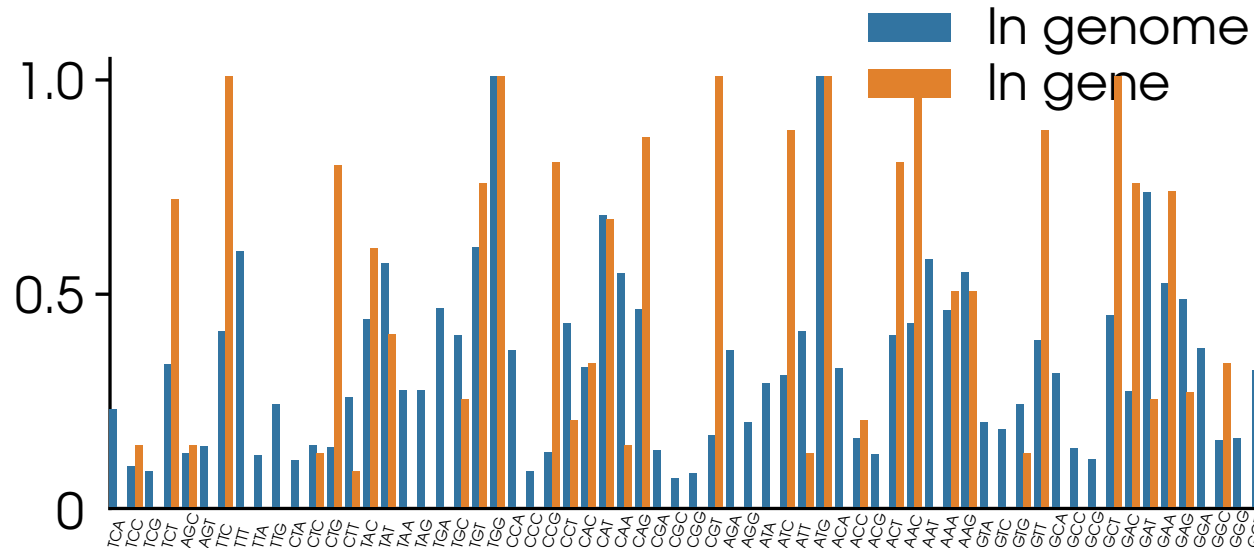

Supplement: Supplementary file 1 [file plants-11-02450-s001.zip › Supplementary_Materials/Figure S1_A.pdf]

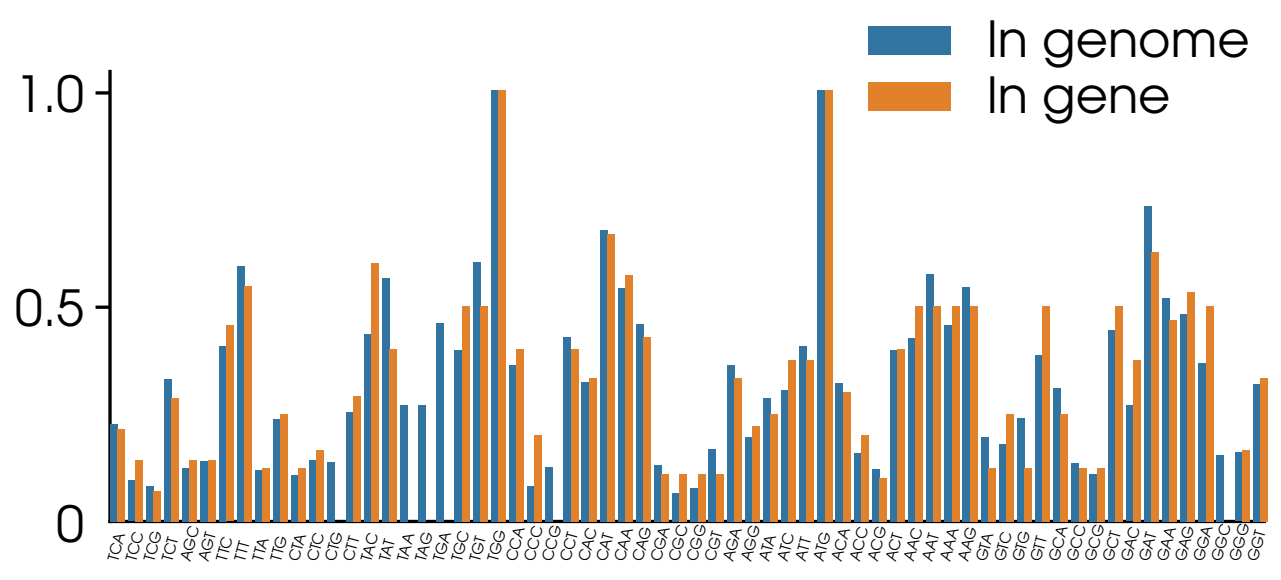

Supplement: Supplementary file 1 [file plants-11-02450-s001.zip › Supplementary_Materials/Figure S1_B.pdf]

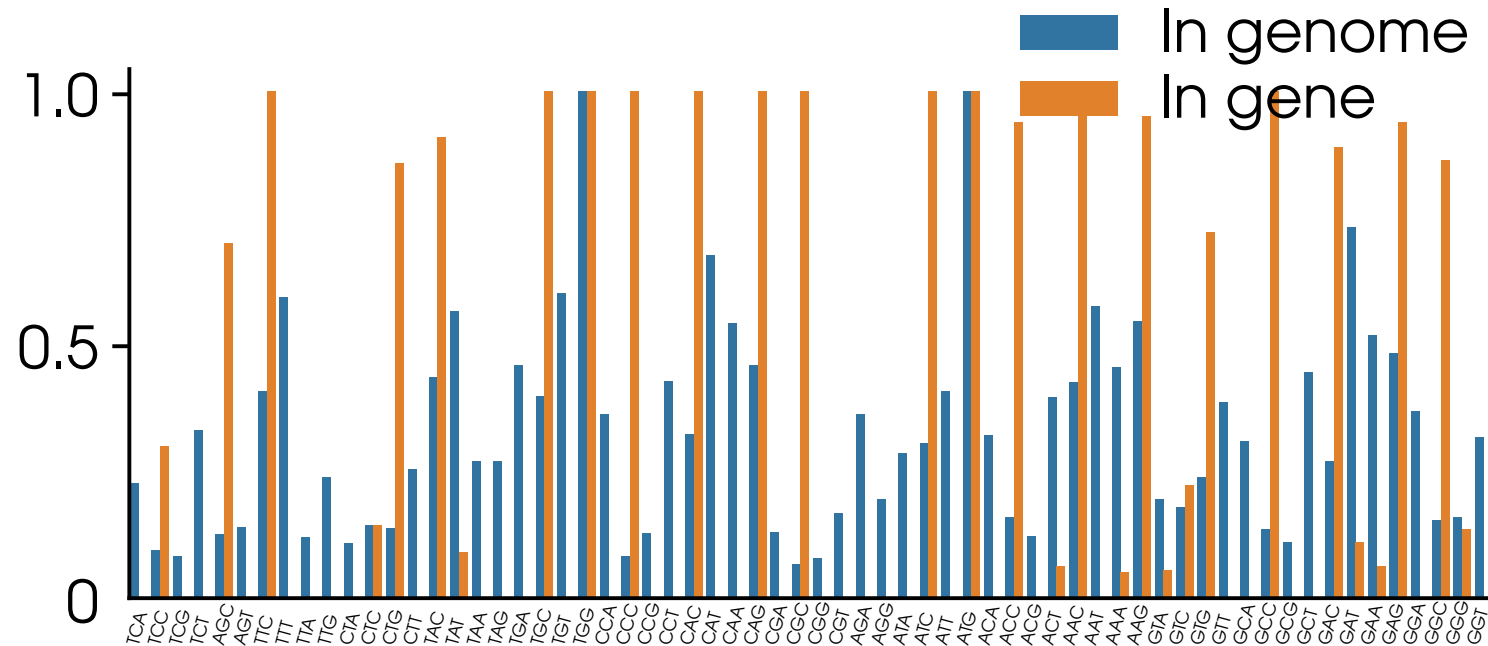

Supplement: Supplementary file 1 [file plants-11-02450-s001.zip › Supplementary_Materials/Figure S1_C.pdf]

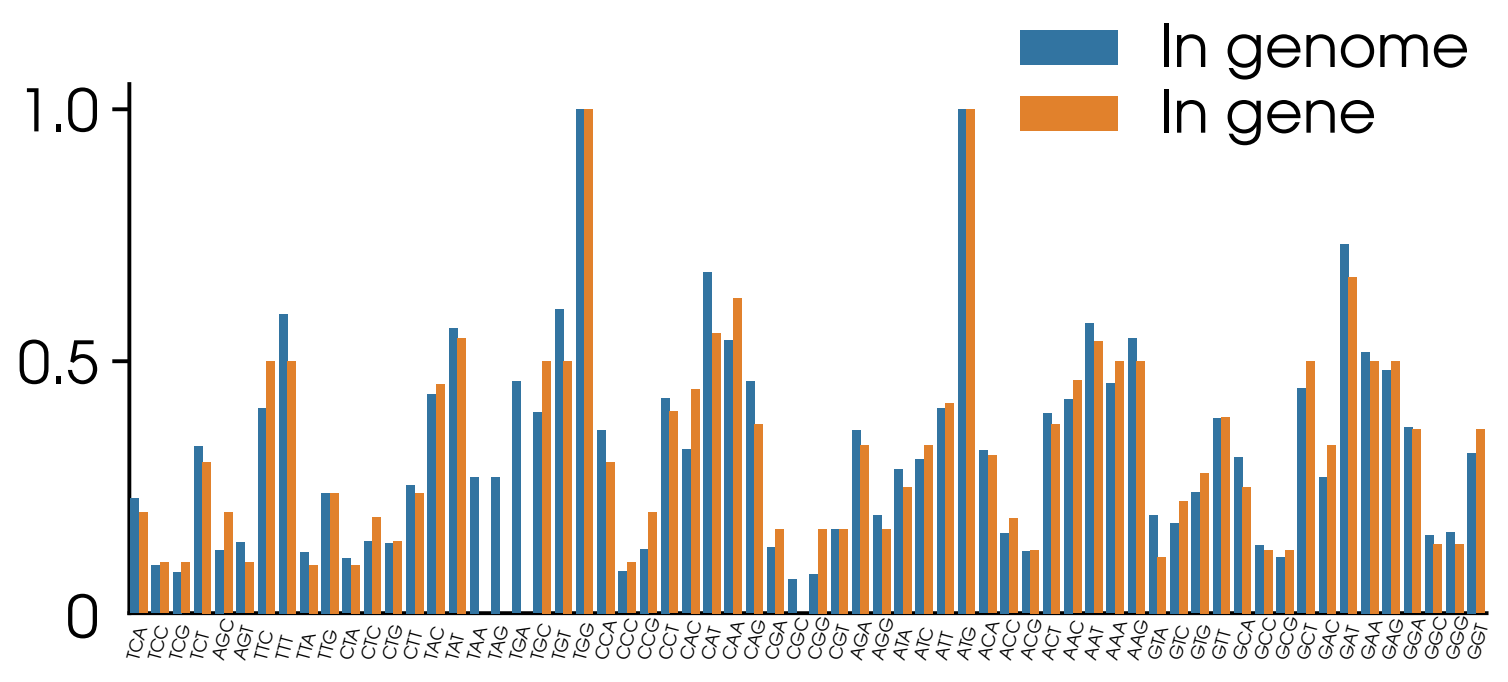

Supplement: Supplementary file 1 [file plants-11-02450-s001.zip › Supplementary_Materials/Figure S1_D.pdf]

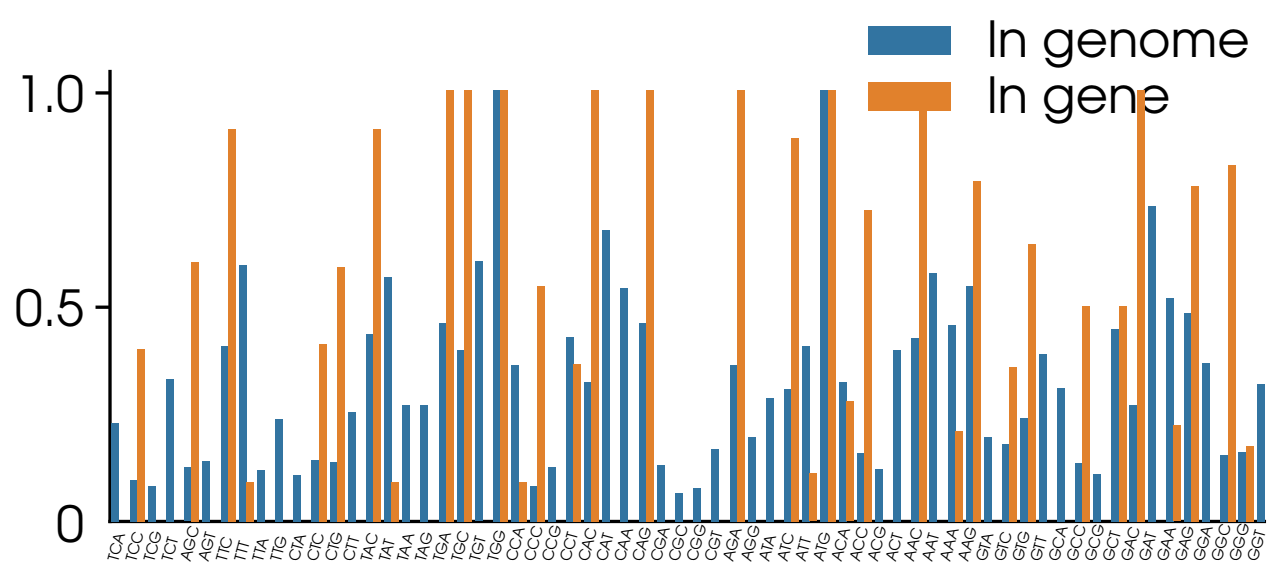

Supplement: Supplementary file 1 [file plants-11-02450-s001.zip › Supplementary_Materials/Figure S1_E.pdf]

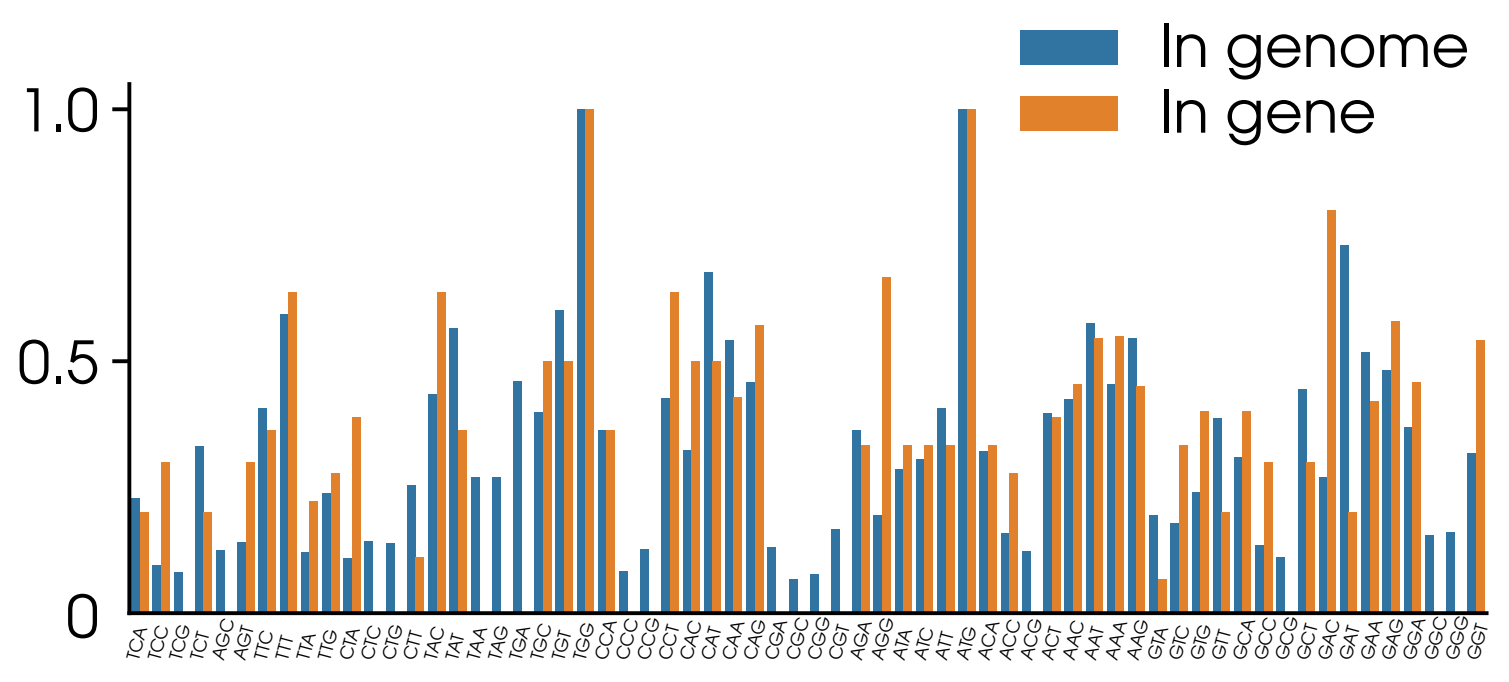

Supplement: Supplementary file 1 [file plants-11-02450-s001.zip › Supplementary_Materials/Figure S1_F.pdf]

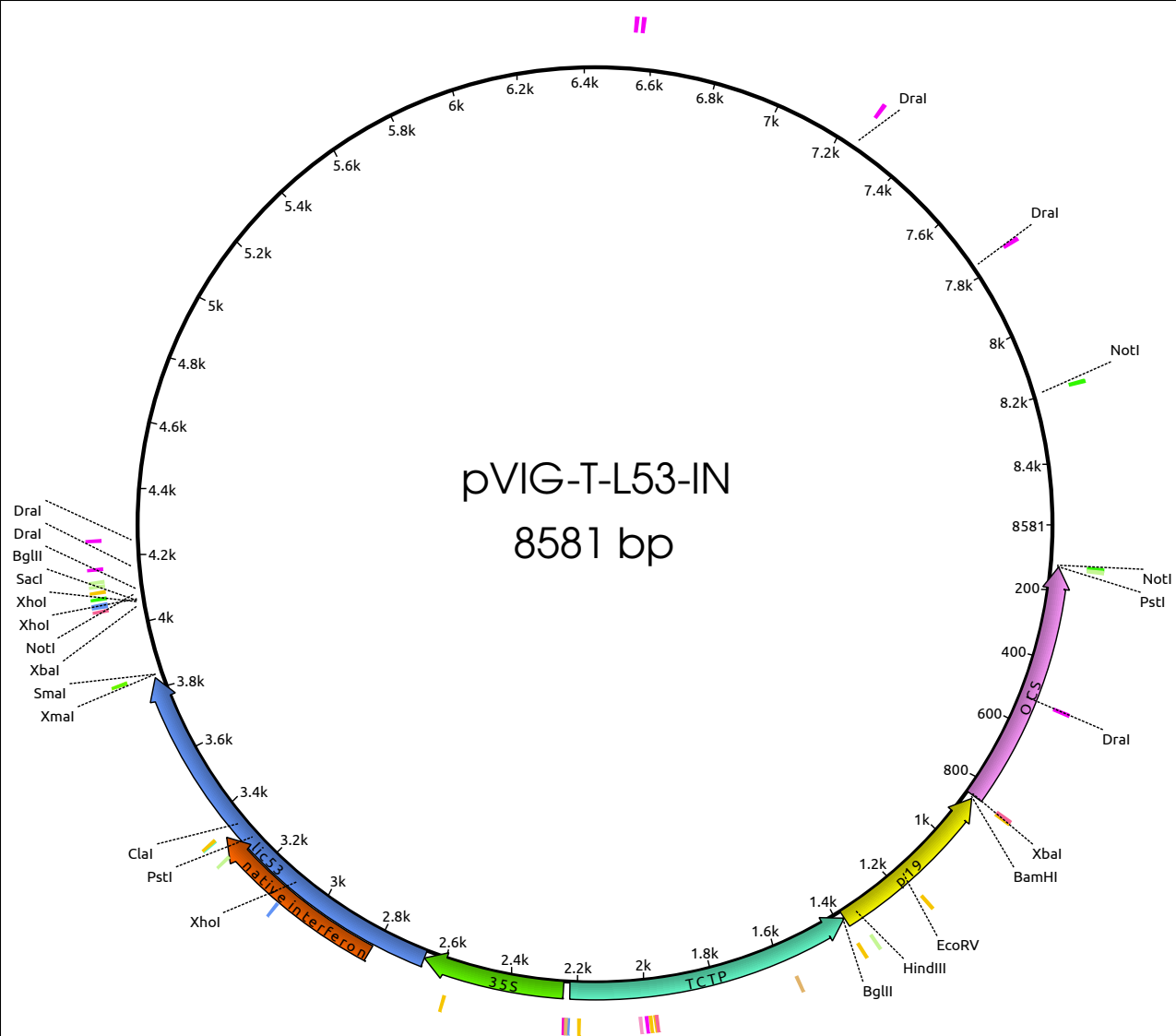

Supplement: Supplementary file 1 [file plants-11-02450-s001.zip › Supplementary_Materials/Figure S2_A.pdf]

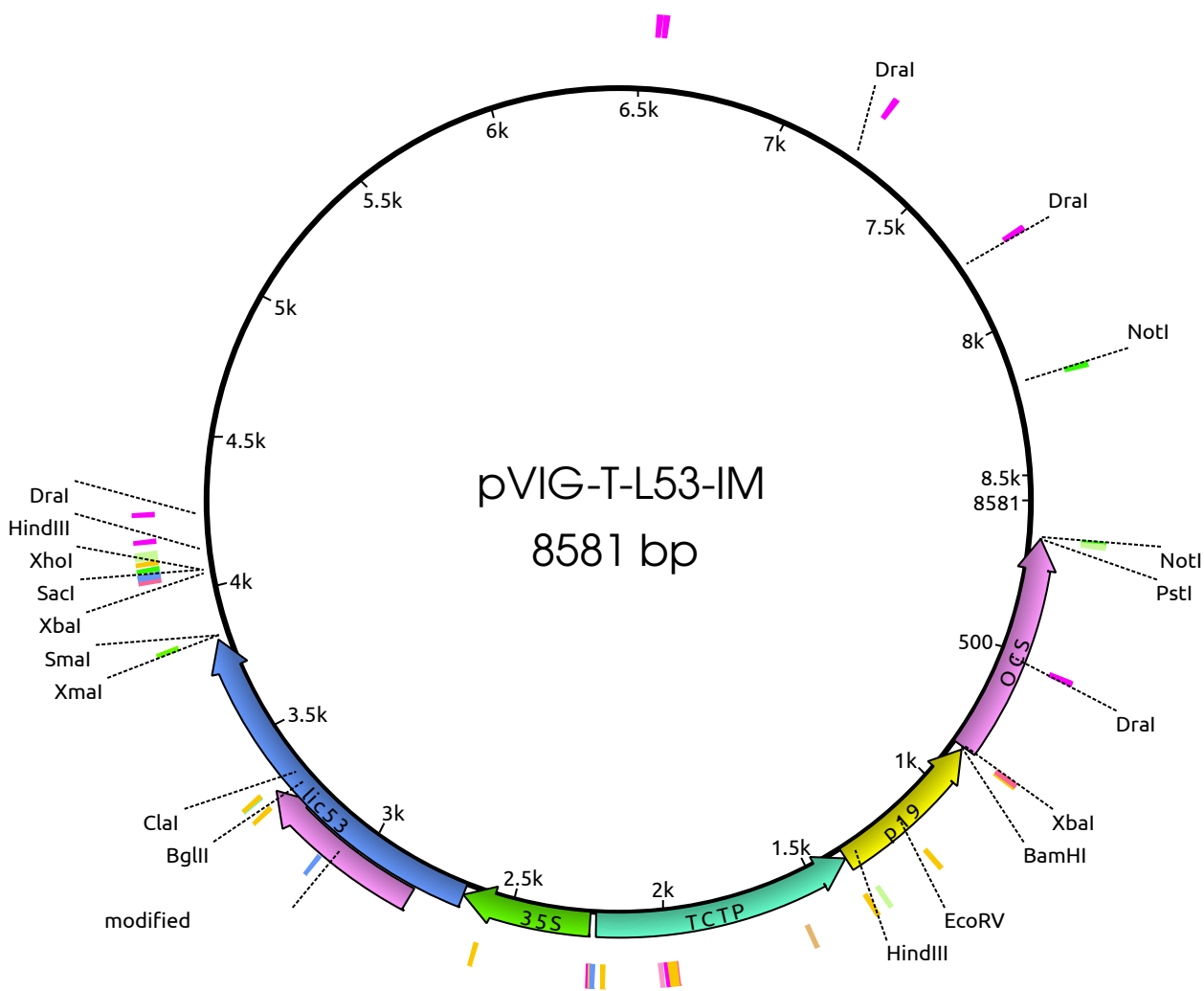

Supplement: Supplementary file 1 [file plants-11-02450-s001.zip › Supplementary_Materials/Figure S2_B.pdf]

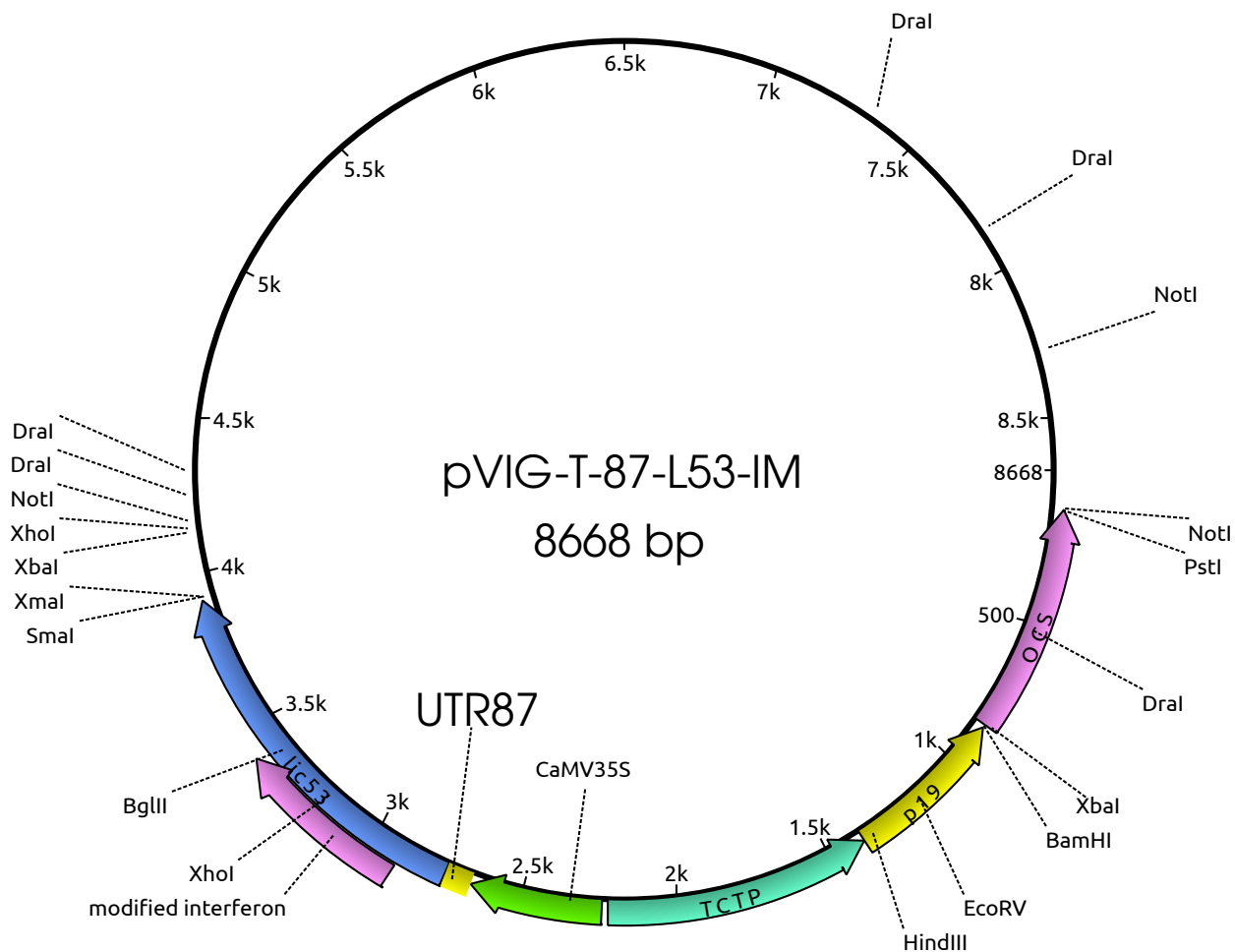

Supplement: Supplementary file 1 [file plants-11-02450-s001.zip › Supplementary_Materials/Figure S2_C.pdf]

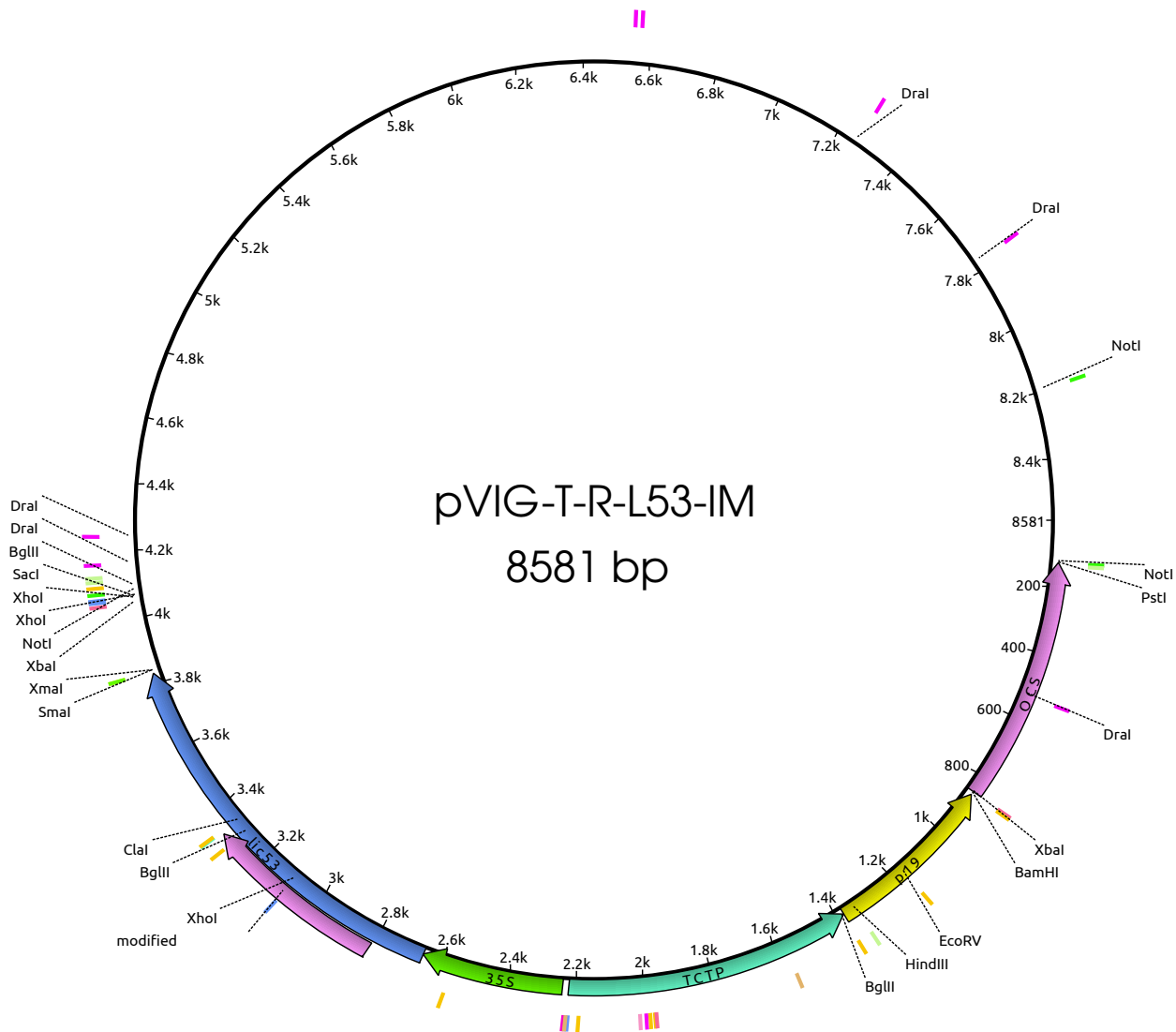

Supplement: Supplementary file 1 [file plants-11-02450-s001.zip › Supplementary_Materials/Figure S2_D.pdf]

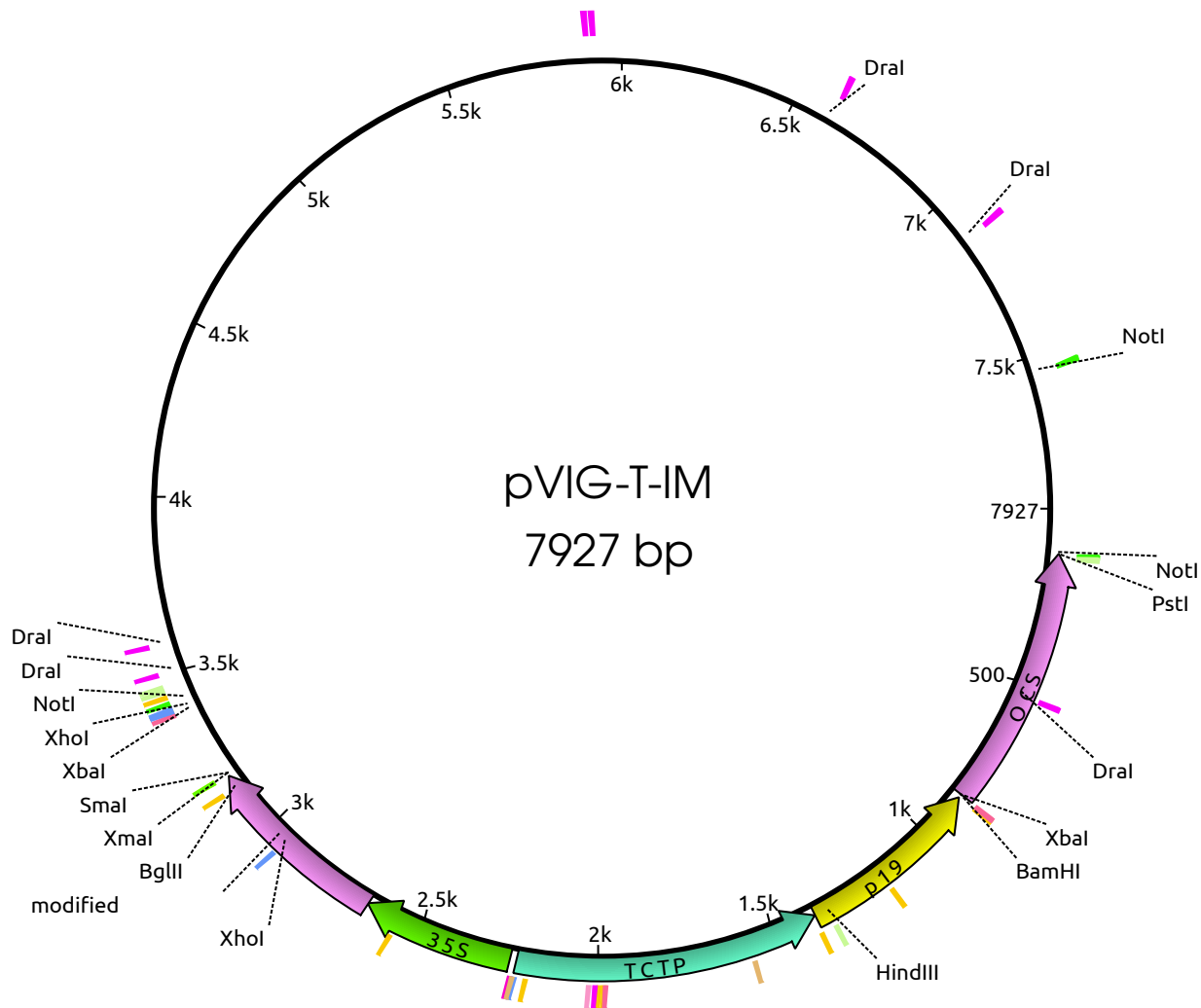

Supplement: Supplementary file 1 [file plants-11-02450-s001.zip › Supplementary_Materials/Figure S2_E.pdf]

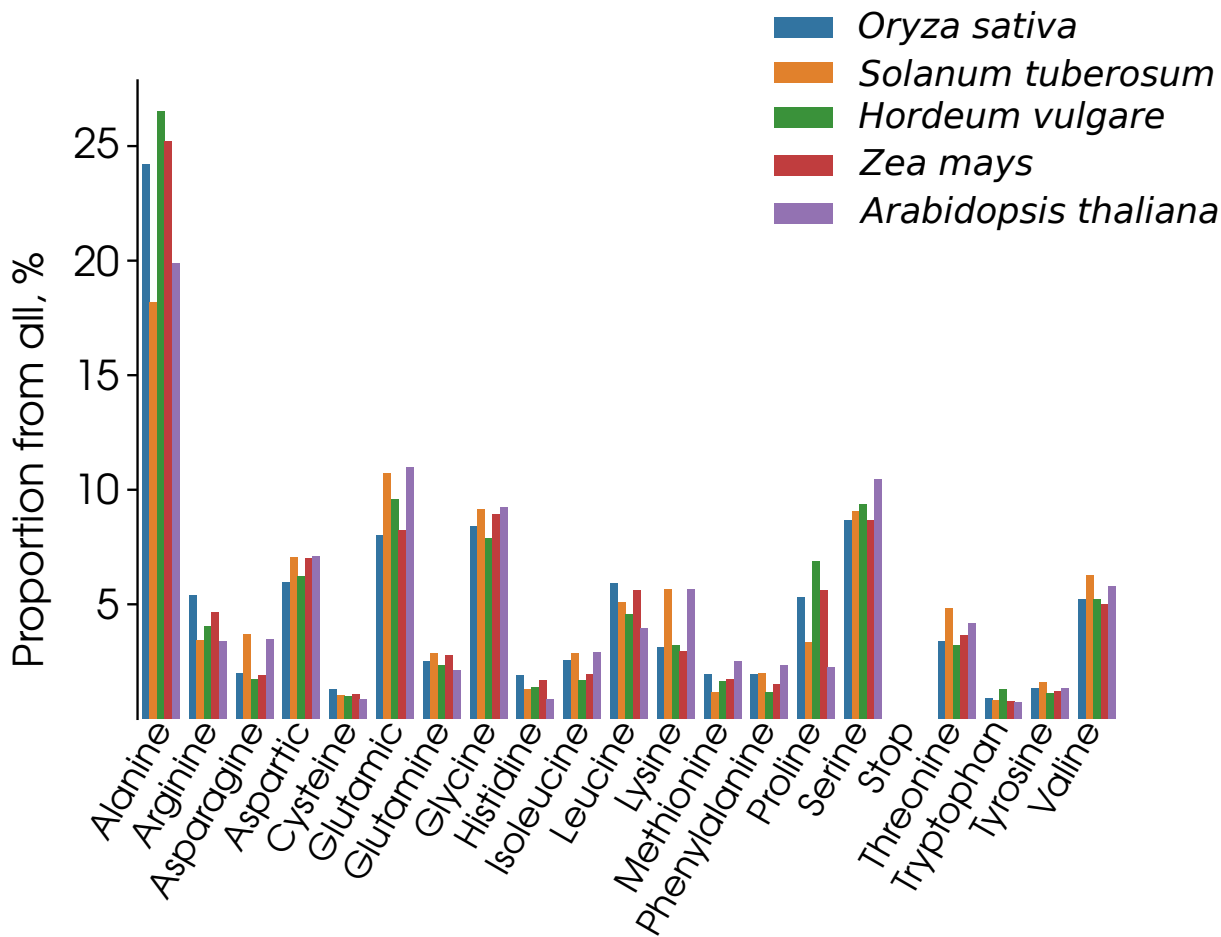

Supplement: Supplementary file 1 [file plants-11-02450-s001.zip › Supplementary_Materials/Figure S3.pdf]

1

2

3

M

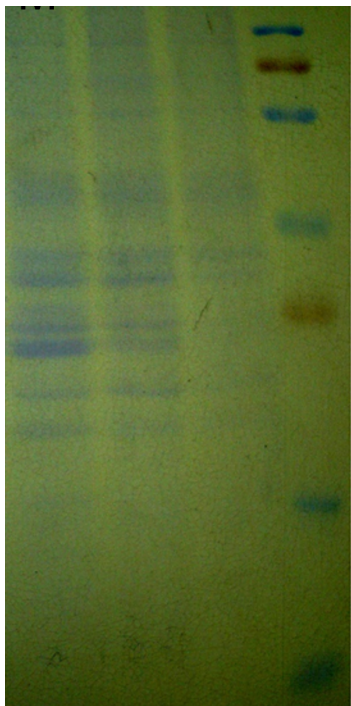

Supplement: Supplementary file 1 [file plants-11-02450-s001.zip › Supplementary_Materials/Figure S4.pdf]
